# A254 HEPATIC SEQUELAE OF POST-ACUTE COVID-19 SYNDROME (PACS): A SYSTEMATIC REVIEW OF THE CURRENT LITERATURE

**DOI:** 10.1093/jcag/gwac036.254

**Published:** 2023-03-07

**Authors:** P Mundra, Z Kailani, M Yaghoobi, S Albashir

**Affiliations:** 1 Medicine, Michael G. DeGroote School of Medicine, McMaster University, Hamilton; 2 Laurier University, Waterloo; 3 Gastroenterology, McMaster University, Hamilton, Canada

## Abstract

**Background:**

Post-acute COVID-19 syndrome (PACS), colloquially known as long-COVID, is a syndrome characterized by persistent or delayed symptoms four weeks or beyond from initial infection onset. Observational studies have shown liver injury during acute COVID-19 infection, particularly in severe infections requiring hospitalization. This has been observed in forms of increased liver enzyme markers, imaging, fibrosis and transplant rejection. However, the data on resolution of abnormal liver injury markers is inconsistent. As such, the long-term hepatic sequelae following acute COVID-19 infection remains unclear.

**Purpose:**

To assess the current understanding of long-term hepatic sequalae of COVID-19 in adults, and to identify prevalence and odds of liver complications in patients with PACS compared to COVID-negative individuals.

**Method:**

Included criteria consisted of observational or cross-sectional studies with patients ≥18y at least four weeks following COVID-19 infection evaluating liver disease/involvement. Liver involvement was defined as per primary study, in order to accomodate various modalities of hepatic injury (e.g. lab markers, imaging, etc.). Case reports or series, systematic reviews, literature reviews, meta-analyses, editorials and commentaries were excluded, as well as studies that report liver function/injury only during acute COVID-19 infection rather than following. Systematic searches were performed on MEDLINE, CINAHL and EMBASE. All studies were screened by two independent reviewers. Risk of bias was assessed by two independent reviewers using the Cochrane ROBIN-E tool. All conflicts from screening, data extraction and risk of bias assessment stages were resolved by a third independent reviewer. Data on biochemical liver markers, imaging, fibrosis and clinical scores were collected where available.

**Result(s):**

2734 studies were found and 614 duplicates were removed. 2117 abstracts and 35 subsequent full texts were screened. Most studies were high or very high risk of bias; only two studies were found to be low risk of bias. Only nine studies utilized control groups. Most studies assessed liver enzymes. 22 of 26 studies suggested some type of abnormality in hepatic testing following COVID-19 infection. 14 studies suggested persistently abnormal liver function tests in PACS patients. 5 studies suggested persistent imaging, fibrosis or autopsy abnormalities. Given the high risk of bias, meta-analysis could not be reliably performed among all included studies.

**Conclusion(s):**

Despite most studies suggesting there is a proportion of patients who suffer from prolonged liver abnormalities such as elevated liver enzymes or fibrosis on imaging, the reliability of these fundings is uncertain given high risk of bias and lack of consistent control groups. As such, there is insufficient high-quality data to inform the natural history of hepatic sequalae following COVID-19 infection. Further high-quality research is required in this field to inform future clinical decisions.

**Please acknowledge all funding agencies by checking the applicable boxes below:**

None

**Disclosure of Interest:**

None Declared

